# Supplementary material for: New Insight into Aspartate Metabolic Pathways in Populus: Linking the Root Responsive Isoenzymes with Amino Acid Biosynthesis during Incompatible Interactions of Fusarium solani
Source: Int J Mol Sci. 2022 Jun 7;23(12):6368. doi: 10.3390/ijms23126368 (PMC9224274; doi:10.3390/ijms23126368)
Supplement: Supplementary file 1 [file ijms-23-06368-s001.zip › ijms-1747228-supplementary.pdf]

New Insight into Aspartate Metabolic Pathways in *Populus*: Linking the Root Responsive Isoenzymes with Amino Acid Biosynthesis during Incompatible Interactions of *Fusarium solani*

Mei Han[1†], Xianglei Xu[1, 2†], Xue Li[1, 2†], Mingyue Xu[1], Mei Hu[1, 3], Yuan Xiong[1, 2], Junhu Feng[1], Hao Wu[1, 2], Hui Zhu[1, 2] and Tao Su[1, 2*]

Supplementary Material

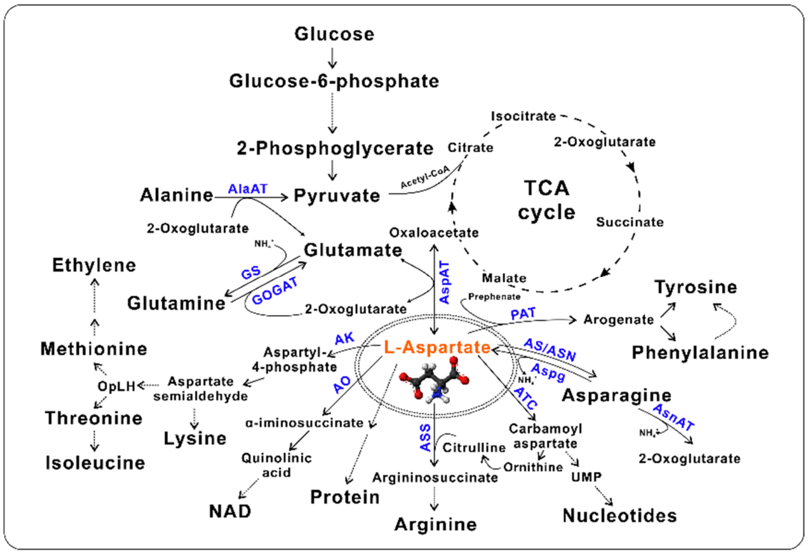

Figure S1. Schematic model representing the central metabolites intermediated by Asp metabolic pathways in plants. PAT, prephenate aminotransferase; TCA cycle, tricarboxylic acid cycle; NAD, nicotinamide adenine dinucleotide; UMP, uridine monophosphate.

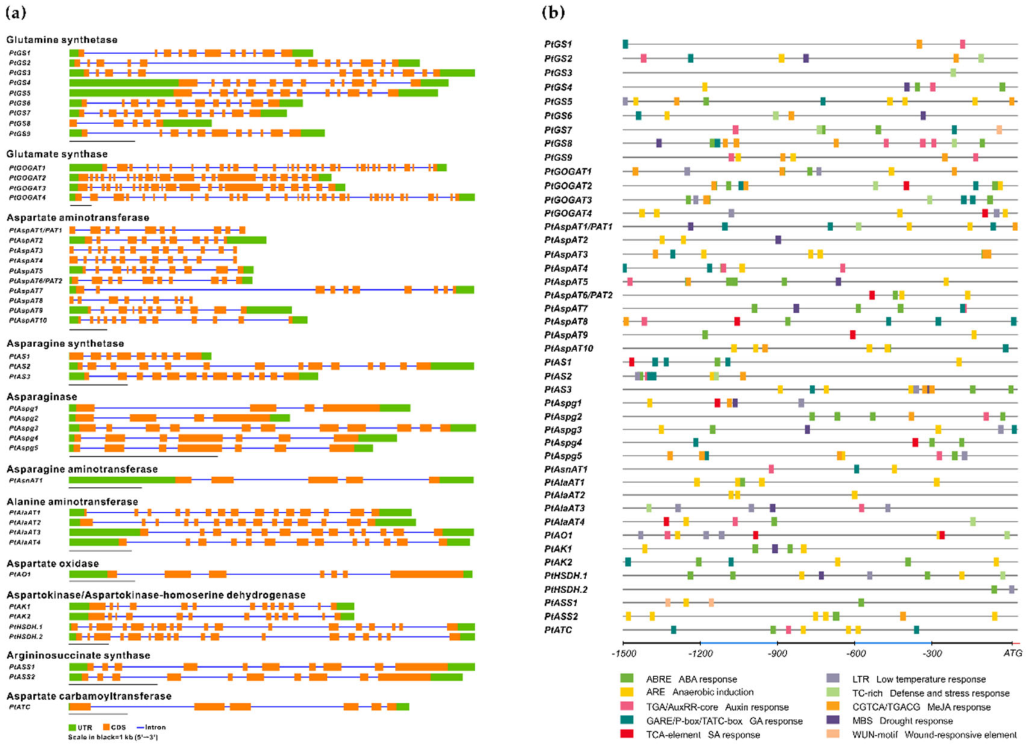

Figure S2. Genomic structures and *in silico* prediction of the *cis*-regulatory elements in gene promoters. (**a**) Genomic structures show the patterns of the exon/intron organization. The exon lengths are displayed proportionally to the scale on the bottom. (**b**) The various *cis*-regulatory elements were boxed in colors.

Table S1. List of isoenzymes involved in aspartate (Asp) metabolic pathways in *P. trichocarpa*.

Enzyme[1]	Gene (v3.1)	Name	Gene (bp)	ORF (bp)	Size (a.a)	MW (kDa)	PI	Target[2]	*A.thaliana*	Name
GLN/GS	Potri.004G085400	*PtGS1/PtGS1.1a*	3321	1071	356	39.26	5.52	Cyto	AT1G48470	*GLN1;5*
	Potri.005G093200	*PtGS2/PtGS1.2a*	4777	1071	356	39.06	5.14	Cyto	AT1G66200	*GLN1;2*
	Potri.007G069600	*PtGS3/PtGS1.2b*	5537	1071	356	38.99	5.53	Cyto	AT3G17820	*GLN1;3*
	Potri.008G200100	*PtGS4/PtGS2a*	5169	1299	432	47.75	6.48	Plastid	AT5G16570	*GLN1;4*
	Potri.010G029100	*PtGS5/PtGS2b*	5029	1299	432	47.89	6.48	Plastid	AT5G35630	*GLN2*
	Potri.012G043900	*PtGS6/PtGS1.3a*	3227	1071	356	39.12	5.86	Cyto	AT5G37600	*GLN1;1*
	Potri.015G034700	*PtGS7/PtGS1.3b*	2964	1071	356	39.22	5.81	Cyto		
	Potri.017G127300*	*PtGS8*	1942	492	163	18.04	8.35	Cyto		
	Potri.017G131100	*PtGS9/PtGS1.1b*	3485	1071	356	39.22	5.95	Cyto		
GLU/GOGAT	Potri.006G038400	*PtGOGAT1*	15954	4476	1491	162.70	5.85	Plastid	AT2G41220	*GLU2*
	Potri.012G011700	*PtGOGAT2*	11095	6687	2228	243.90	6.48	Plastid	AT5G04140	*GLU1*
	Potri.015G017500	*PtGOGAT3*	11684	6687	2228	244.10	6.15	Plastid	AT5G53460	*GLT1*
	Potri.016G036900	*PtGOGAT4*	17137	4863	1620	176.53	6.41	Plastid		
AspAT	Potri.005G079200	*PtAspAT1/PAT1*	4021	1443	480	51.59	7.11	Plastid	AT1G62800	*ASP4*
	Potri.006G107100	*PtAspAT2*	4501	1269	422	46.96	7.19	Mito	AT2G22250	*PAT*
	Potri.006G241500	*PtAspAT3*	3832	1266	421	46.56	6.50	Cyto	AT2G30970	*ASP1*
	Potri.006G241600	*PtAspAT4*	3829	1350	449	49.18	9.16	Cyto	AT4G31990	*ASP5*
	Potri.006G260200	*PtAspAT5*	4211	1401	466	51.03	8.67	Plastid	AT5G11520	*ASP3*
	Potri.T079800**	*PtAspAT6/PAT2*	4159	1446	481	51.89	8.06	Plastid	AT5G19550	*ASP2*

	Potri.014G143300	*PtAspAT7*	9230	1287	428	47.79	8.35	Mito		
	Potri.016G133000*	*PtAspAT8*	2806	915	304	33.11	5.66	Cyto		
	Potri.018G022200	*PtAspAT9*	5060	1401	466	50.92	8.67	Plastid		
	Potri.018G082500	*PtAspAT10*	5417	1224	407	44.47	7.72	Cyto		
AS/ASN	Potri.001G278400	*PtAS1*	2272	1338	445	49.77	6.58	Cyto	AT3G47340	*ASN1*
	Potri.005G075700	*PtAS2*	6490	1755	584	65.64	6.39	Cyto	AT5G10240	*ASN3*
	Potri.009G072900	*PtAS3*	3969	1770	589	66.07	6.00	Cyto	AT5G65010	*ASN2*
Aspg	Potri.002G122900	*PtAspg1*	3038	987	328	34.68	4.69	Cyto	AT3G16150	*ASPGB1*
	Potri.014G022900	*PtAspg2*	1967	987	328	34.60	4.81	Per	AT5G08100	*ASPGA1*
	Potri.014G080600	*PtAspg3*	3622	1260	419	43.71	6.03	Cyto		
	Potri.014G191600	*PtAspg4*	2919	1077	358	37.90	6.14	ER		
	Potri.014G191900	*PtAspg5*	2705	1077	358	37.93	6.14	Extra		
AsnAT	Potri.001G253300	*PtAsnAT1*	5120	1206	401	44.08	8.59	Per	AT2G13360	*AGT1*
AlaAT	Potri.001G162800	*PtAlaAT1*	5087	1446	481	52.98	6.25	Per	AT1G17290	*ALAAT1*
	Potri.003G072600	*PtAlaAT2*	5155	1602	533	58.27	5.77	Mito	AT1G72330	*ALAAT2*
	Potri.008G187400	*PtAlaAT3*	6013	1446	481	53.37	5.99	Per	AT1G23310	*GGT1*
	Potri.010G045100	*PtAlaAT4*	5953	1446	481	53.48	6.92	Per	AT1G70580	*GGT2*
AO	Potri.001G348600	*PtAO1*	5603	1953	650	71.70	6.40	Plastid	AT5G14760	*AO*
AK/AK-HSDH	Potri.002G236800	*PtAK1*	6690	1674	557	61.05	5.72	Plastid	AT3G02020	*AK3*
	Potri.014G145700	*PtAK2*	6860	1686	561	61.71	5.69	Plastid	AT5G13280	*AK1*
	Potri.013G099500	*PtHSDH.1*	9727	2769	922	100.80	6.94	Plastid	AT5G14060	*AK2*
	Potri.019G071700	*PtHSDH.2*	9535	2751	916	100.33	6.70	Plastid	AT1G31230	*HSDH1*
									AT4G19710	*HSDH2*
ASS	Potri.008G020200	*PtASS1*	4278	1485	494	54.25	6.31	Plastid	AT4G24830	*ASS*
	Potri.010G239000	*PtASS2*	4140	1485	494	54.35	6.45	Plastid		
ATC	Potri.001G357200	*PtATC*	5200	1155	384	42.94	6.78	Plastid	AT3G20330	*ATC*

[1] glutamine synthetase (GLN/GS), glutamate synthase (GLU/GOGAT), Asp aminotransferase (AspAT), asparagine synthetase (AS/ASN), asparaginase (Aspg), asparagine aminotransferase (AsnAT), alanine aminotransferase (AlaAT), Asp oxidase (AO), aspartokinase/aspartokinase-homoserine dehydrogenases (AK/AK-HSDH), argininosuccinate synthase (ASS), Asp transcarbamoylase/carbamoyltransferase (ATC).

[2] cytoplasm (Cyto), extracellular (Extra), mitochondrion (Mito), plastid, endoplasmic reticulum (ER), and peroxisome (Per). * Genes ID only present in v3.0, ** The ID is updated to Potri.007G088426 in v4.1.

Table S2. The number of annotated compounds classified by families upon *F. solani* infection in roots.

Chemical Family	Number of annotated compounds		
	**48/0 hpi**	**72/0 hpi**	**72/48 hpi**
Carbohydrates and carbohydrate conjugates	70	56	15
Flavonoid glycosides	61	38	7
Triterpenoids	45	13	11
Terpenoids (mono, di, and sesqui)	28	21	4
Amino acids, peptides, and analogues	26	24	9
Flavans	18	8	5
Fatty acid-related	13	16	5
Stilbene glycosides	12	10	1
Biflavonoids and polyflavonoids	11	12	3
Furanocoumarins	9	5	1
Hydroxycinnamic acids and derivatives	8	8	1
Amines	6	6	1

Unclassified	158	133	26

Table S3. Statistical list of variable annotated amino acids and derivatives.

Compound	KEGG/PubChem ID	log2FC (72/0 hpi)	log2FC (48/0 hpi)	log2FC (72/48hpi)
3-amino-2-piperidone		-0.959242085	-1.287624277	ND
Acetylhomoserine		0.413502763	0.289123839	ND
Citrulline	C00327	ND	-0.965678271	0.72762098
gamma-Glutamylaspartic acid		0.811968258	0.980476886	ND
Glycine		0.93688711	0.9762432	ND
Glycylglycylglycine		12.07483783	ND	3.245442267
L-2-Amino-5-hydroxypentanoic acid		1.31739572	1.134765593	ND
L-alpha-Aminobutyric acid	C02356	2.484660714	1.96971639	ND
L-Asparagine	C00152	-1.39960787	-1.040911987	ND
L-asparagine		-2.243842063	-1.406524372	ND
L-Glutamic acid	C00025	0.871826199	0.70855538	ND
L-Isoleucine	C00407	ND	0.347996561	-0.695894603
L-Oxalylalbizziine		0.580167883	0.415950772	ND
L-proline		1.334475436	1.356375227	ND
L-threonine		ND	0.82296079	-0.535254662
Lycoperdic acid		1.381155267	1.455909502	ND
Ornithine		ND	-1.234841524	0.559956343
S-Methylmethionine		5.031482626	ND	1.952267173

Table S4. List of primers used for qRT-PCR analyses.

Primer Name	Sequence	Access number/Gene
β-Actin_qF	GACCTTCAATGTGCCTGCAA	Potri.019G006700.1/*Ptβ-Actin*
β-Actin_qR	ACCATCACCAGAATCCAGCA	
EF1α_qF	TCCGTCTTCCACTTCAGGATGTCT	Potri.006G130900.1/*PtEF1α*
EF1α_qR	GTCACGACCATACCAGGCTTCAG	
UBIC_qF2	TTGCTTGAGGAACTTGAAC	Potri.006G205700.7/*PtUBIC*
UBIC_qR2	GTGAGGACCGATTACAGT	
qFW113	CGCATCAGCTATAAAAGAAGCAC	Potri.004G085400/*PtGS1*
qRV113	TCTCAGTGGAGTCTGAGAGGTT	
qFW114	AAATGCCAGTGTTGGCTGTTG	Potri.005G093200/*PtGS2*
qRV114	TGAAAAATGCAGAGAATTGTGACT	
qFW116	CTCCTTGACTTTGGGGATGC	Potri.007G069600/*PtGS3*
qRV116	TCCAAGTCAATGCCACCTTAT	
qFW117	CCCGTGGATGAAAAGGGTCT	

qRV117	TTCCACACAAGGCCAAGGAA	Potri.008G200100/*PtGS4*
qFW118	ATCAGCATAACAAGGGCGACA	Potri.010G029100/*PtGS5*
qRV118	CCCCGTGTTTGGGGATTAAGA	
qFW119	TTCAGCTGTACCTTCGAGCC	Potri.012G043900/*PtGS6*
qRV119	TCGACGGTGAAGGCGTTTTA	
qFW120	ATGATTGTCCTCCGTTCCCTG	Potri.015G034700/*PtGS7*
qRV120	CTACCAAACACACCCACCCTT	
qFW121	AGCGTGGTCTGAGGCATATC	Potri.017G127300*/*PtGS8*
qRV121	CGATTAGCAACACCCCGTTTG	
qFW122	TCTTCAACAATAATCCGCCCT	Potri.017G131100/*PtGS9*
qRV122	AAGGTCTGAAAGCAACGACAT	
qFW123	GTGCATCATTCGAGGCAACC	Potri.006G038400/*PtGOGAT1*
qRV123	CGAATTCGGGTTTTTCCGGC	
qFW124	TGCTTATGAGCGTGAAGGTGT	Potri.012G011700/*PtGOGAT2*
qRV124	AGGGCATCCCGAGTTTTCTC	
qFW125	GCTCATTTGGTAGTTGTTGAGGA	Potri.015G017500/*PtGOGAT3*
qRV125	GCTAAACCACAAGGCACAACG	
qFW126	ACATCACCCAAGAGCATCCC	Potri.016G036900/*PtGOGAT4*
qRV126	GTCCTGGGTGTGTTCAGCAT	
PtAspAT1_qFw	GCAGAGGTTGAAGGGTTTGG	Potri.005G079200/*PtAspAT1*/*PAT1*
PtAspAT1_qRv	TGTAGGGTGGTCAGAGAAGC	
PtAspAT2_qFw	TCCAACCACCATAACATA	Potri.006G107100/*PtAspAT2*
PtAspAT2_qRv	CATTCTTGACATCATCCAT	
PtAspAT3_qFw	GGACAATGAGCCCACGAATTG	Potri.006G241500/*PtAspAT3*
PtAspAT3_qRv	TCCTCTATTCTCCTCCACCACTT	
PtAspAT4_qFw	GTTGCAGGCAGGGTTGAAAG	Potri.006G241600/*PtAspAT4*
PtAspAT4_qRv	GGCTTCAAAGAGTTTCTGGCG	
PtAspAT5_qFw	TGCACGTGGTATGGAGCTTT	Potri.006G260200/*PtAspAT5*
PtAspAT5_qRv	TCGGGCAATCCTTTTCAGCT	
PtAspAT6_qFw	TAGCAATGGTGCCAAGCAGA	Potri.T079800/*PtAspAT6*/*PAT2*
PtAspAT6_qRv	TCACCCAAAATGGTGCAGGA	
PtAspAT7_qFw	AGCAATCCTCCTGTTCAT	Potri.014G143300/*PtAspAT7*
PtAspAT7_qRv	CAGCCATTACCTTCACTTC	
PtAspAT8_qFw	GAAGATGGGCACCTGGTAGG	Potri.016G133000*/*PtAspAT8*
PtAspAT8_qRv	GCTTTCCCGCAAAGTTGTCC	
PtAspAT9_qFw	TCCTCCTGTTCATGGGGCTA	Potri.018G022200/*PtAspAT9*
PtAspAT9_qRv	CCTCCCAGCCATCATTTCCA	
PtAspAT10_qFw	CACTGGCTTGAACACTGAACA	Potri.018G082500/*PtAspAT10*
PtAspAT10_qRv	AAAAGATCCCCAGAAGCGGA	
qFW127	CCGTTCTCTTGATTTTTCAGGGTC	Potri.001G278400/*PtAS1*
qRV127	GTGGGTCATATGGGGTCGAG	
qFW128	GTGCCCCTCTCCTGTGTTTT	Potri.005G075700/*PtAS2*
qRV128	AGATGGGTGAATGATGCTCGT	
qFW129	AAGCCTTTGACGACGAGGAG	Potri.009G072900/*PtAS3*
qRV129	TGTCAGTCACATGTTGGGCA	
qFR130	TGTCAAGTCGGAGCACAATCT	

qRV130	AAATTCTTTCCTTGTCGCGGG	Potri.002G122900/*PtAspg1*
qFW132	GATGGGCTATAGCGGTGCAT	Potri.014G022900/*PtAspg2*
qRV132	CGAAGAGCAGAGATGCCGAT	
qFW133	GAAGCCAAAAGTCCCAAGCG	Potri.014G080600/*PtAspg3*
qRV133	CCAGGACCCTTGCGAAGAAT	
qFW134	TTGGAGGTCCCACTGGAATG	Potri.014G191600/*PtAspg4*
qRV134	CTTATCGAAGTTTTTAGTCGTGCT	
qFW135	GACCAGAGCAGAACGGAACA	Potri.014G191900/*PtAspg5*
qRV135	AAAGGCCAGGTGCTCACTAC	
qFW136	TGAGACTCCCAAGGTCTCGT	Potri.001G253300/*AsnAT1*
qRV136	TCTGCATCAGAAGTACCTGTC	
qFW213	ACGCATGGTTTGTGGCATTG	Potri.001G162800/*PtAlaAT1*
qRV213	TCCTACCACCTTCGAAAGCC	
qFW214	AATAATACACGCCCCCAGGC	Potri.003G072600/*PtAlaAT2*
qRV214	TTCAAGGAACTGACGAGCCG	
qFW215	AGTTGCCGATGGACCTAAGC	Potri.008G187400/*PtAlaAT3*
qRV215	AAGCCCTTAGAGGAGGTCGT	
qFW216	TGCAAGCTGTTTCGTGTATGC	Potri.010G045100/*PtAlaAT4*
qRV216	AAGCTTGAGCTTTCTTCACCT	
qFW217	GGGGGCAAGAAAAAGGCAC	Potri.001G348600/*PtAO1*
qRV217	TGATCTCGAGGAGCAACGG	
qFW218	AGCCAAGTTTGGACTGATCG	Potri.002G236800/*PtAK1*
qRV218	CGGACCAGTAAAGTGACCCA	
qFW219	TGATACAGCCATCTCATCGTG	Potri.014G145700/*PtAK2*
qRV219	AACAATGGCCCAAAGGTTAGA	
qFW220	TCAAAGCAAGGGCTGTCTGC	Potri.013G099500/*PtHSDH.1*
qRV220	TAGGAGAGGGTCCGCTAGGA	
qFW221	CGCGAGCATTTAGTGACGTG	Potri.019G071700/*PtHSDH.2*
qRV221	GCTCTCAGTGGTTGAGGGAC	
qFW222	CAGCGGATGTTTTGTCAAGCA	Potri.008G020200/*PtASS1*
qRV222	GGCCTCCTGGTGAGAGATTG	
qFW223	TGACAAGCAGGGTACTTGGG	Potri.010G239000/*PtASS2*
qRV223	CCACATGCACAAGAATCCGA	
qFWATC	GGTCCTGGACAACATCCCAC	Potri.001G357200/*PtATC*
qRVATC	GCGGACTGTCCTCCCATTAG	
